# Matrine Protects Intestinal Barrier Function via MicroRNA-155 Through ROCK1-Signaling Pathway

**DOI:** 10.5152/tjg.2023.21884

**Published:** 2023-08-01

**Authors:** Dongdong Yu, Dan Su, Zhihua Liu

**Affiliations:** 1 Department of AnoRectal Surgery, The Tenth Affiliated Hospital of Southern Medical University (Dongguan People’s Hospital), Dongguan, Guangdong, China; 2 Department of AnoRectal Surgery, the Sixth Affiliated Hospital of Sun Yatsen University, Guangzhou, Guangdong, China; 3 Key Laboratory of Biological Targeting Diagnosis, Therapy and Rehabilitation of Guangdong Higher Education Institutes, The Fifth Affiliated Hospital of Guangzhou Medical University, Guangzhou, Guangdong, China

**Keywords:** Matrine, microRNA-155, intestinal barrier, ROCK1, occludin, tight junction

## Abstract

**Background/Aims:**

The aim of this study was to investigate the protective effects on intestinal barrier function and tight junction by matrine via a small, non-coding RNA microRNA-155-signaling pathway.

**Materials and Methods:**

Tight junction protein and target gene expressions were determined through microRNA-155 inhibition or overexpression of Caco-2 cell line with or without the treatment of matrine. In order to further verify the role of matrine, dextran sulfate sodium-induced mice colitis was treated by matrine. MicroRNA-155 and ROCK1 expressions were detected in clinical specimens of acute obstruction patients.

**Results:**

Matrine could enhance the expression level of occludin, which might be inhibited by the overexpression of microRNA-155. After the transfection of the precursor of microRNA-155 into Caco-2 cells, ROCK1 expression was increased at both mRNA and protein levels. MicroRNA-155 inhibitor could decrease the ROCK1 expression after transfection. Furthermore, matrine could increase the permeability and decrease tight junction-associated proteins induced by dextran sulfate sodium-induced mice colitis. Clinical sample detection also found high levels of microRNA-155 in stercoral obstruction patients.

**Conclusions:**

Matrine could maintain the tight junction and protect the intestinal barrier from dysfunction. The molecular mechanism may be that matrine might inhibit microRNA-155 and increase the expression level of tight junction proteins.

**Main Points**

- Matrine could protect the intestinal tight junction.
- Matrine could inhibit miR-155.
- miR-155 is associated with the expression of intestinal tight junction.

## Introduction

MicroRNAs (miRs) can bind to the 3ʹ-untranslated regions of mRNA and negatively regulate their target genes. A series of studies have shown that miRs could regulate the expression of different genes.^1,2^ Several hundred miRs have been described, most of which are encoded by the human genome.^3^ MicroRNAs could regulate different genes in a variety of biological processes, such as proliferation, differentiation, and metabolism.^4^ Recently, miRs are also found to be related with tight junction (TJ) and intestinal barrier dysfunction.^5^

Matrine is a natural alkaloid with bioactive components of herbs, such as *Sophora flavescens* Ait.^6^ It possesses a variety of pharmacological characteristics, such as anti-inflammatory and antioxidant characteristics.^7^ The normal use of matrine includes its antitumor, antiviral, and anti-inflammatory effects. As to the side effects, if the infusion speed of matrine is too fast, sometimes it might cause dizziness, nausea, and other adverse reactions. The protective effects of matrine on interleukin-10 (IL-10)-deficient colitis mice have been demonstrated,^8^ while other studies found its effects on mice colitis induced by 2,4,6-trinitrobenzene sulfonic acid.^9,10^ Another study investigated the model of dextran sulfate sodium (DSS)-induced colitis and found that matrine protects against murine colitis by improving gut barrier integrity through the peroxisome proliferator-activated receptor-α (PPAR-α)-signaling pathway as well as the regulation of gut microbiota.^6^

The study about miRNA, intestinal barrier dysfunction, and intestinal microbiota becomes more and more popular.^11,12^ Little further functional characterization was reported to verify the relationship.^13-15^ McKenna et al^16^ investigated the miRNA expression profile using the model of intestinal conditional mutant mice and found intestinal barrier dysfunction in the Dicer1-deficient mice as well as the intestinal inflammation. Liu et al^17^ found that miR-155 antagomir could relieve weight loss and intestinal damage in DSS-induced colitis. Another study showed that FG-4497 might upregulate hypoxia-inducible factor (HIF)-1α expression and protect the intestinal barrier and TJ from dysfunction in DSS-induced colitis model. In our previous study, we identified ROCK1 as the target of miR-21 and demonstrated that miR-21 could protect the intestinal barrier via the Rho-ROCK-signaling pathway of ROCK1.^18^ The general therapeutic method for intestinal barrier dysfunction is using probiotics. Also nowadays, fecal microbiota transplantation is developed for the protection of intestinal barrier.

However, the molecular mechanism and signaling pathway of matrine on colitis have not been fully understood. The relationship between matrine and miR-155 still needs to be further investigated. In this study, we are to investigate the molecular mechanisms and signaling pathway of intestinal epithelial barrier dysfunction, which could be protected by matrine through miR-155.

## Materials and Methods

### Cell Culture

Caco-2 cells were grown and cultured in Dulbecco’s Modified Eagle Medium (DMEM) medium, which was supplemented with 10% Fetal Bovine Serum (FBS), 100 U/mL penicillin, and 100 μg/mL streptomycin at 37°C in a proper humidified atmosphere containing 5% CO_2_. Cells were passaged at pre-confluent densities using 0.05% trypsin and 0.5 mm ethylene diamine tetraacetic acid (EDTA) (Gibco, Carlsbad, CA, USA), as previously described.^19^ Matrine was purchased from the Chengdu Must Bio-Technology Co., Ltd, ChengDu, Sichuan, China.^6^

### Transfection of Caco-2 cells with MicroRNA-155 Overexpression of Lentivirus

As our previous study reported,^18,20^ primers for miR-155, GTCGTATCCAGTGCAGGGTCCGAGGTATTCGCACTGGATACGACACCCCT, were added to the reverse transcriptase reaction mix.^21^ Lentivirus vectors or inhibitors were constructed by Shanghai Minzai Co., Ltd. (Shanghai, China). According to the manufacturer’s instructions, the Caco-2 cells were transfected with Lipofectamine 2000 (Invitrogen, Vilnius, Lithuania).

### Dextran Sulfate Sodium-Induced Mice Colitis

As described,^17^ 3.0% DSS (36-50 kDa; MP Biomedicals, Santa Ana, California, USA) was added in the drinking water of mice for 7 days to induce colitis. Animals were sacrificed after blood was collected from their eyeballs. Mice in the matrine groups were given 20 mg/kg matrine via oral gavage once a day, respectively.^6^

### Permeability Assay

To evaluate the permeability of Caco-2 cells and DSS-induced mice colitis, measurements of transepithelial electrical resistance (TER) and dextran permeability were done, as reported previously.^19^ The fractional excretion (for sucralose) was determined to evaluate the colonic permeability, while the ratio of fractional excretion (for lactulose/mannitol) was used to detect the small intestinal permeability. Ussing chamber assay was also an indicator to evaluate the intestinal permeability, using the isolated mice colons, as previously reported.^19^

### Quantitative Real-Time Polymerase Chain Reaction Analysis

The miR isolation of Caco-2 cells, colon tissues, or serum samples was performed, using the miRNeasy Mini Kit (Qiagen, Hilden, Germany) according to the manufacturer’s instructions. First-strand synthesis of cDNA was performed with the Reverse Transcription Kit (Invitrogen). A quantitative real-time polymerase chain reaction (qRT-PCR) for mRNA was performed through the SYBR Master Mix (Invitrogen) according to the manufacturer’s instructions and a 7500 real-time PCR system. Quantitative real-time polymerase chain reaction primer sequences are shown in Table 1.

### Western Blot Analysis

Western blotting was performed by a wet electroblotter (Bio-Rad, Hercules, CA, USA) for 120 minutes at 100 V, as previously reported.^12,18^ The membrane was detected by the enhanced chemiluminescence method (ECL kit; Pierce, Ill, USA) according to the manufacturer’s instructions.^11^

### Statistical Analysis

Statistical data were analyzed by GraphPad Prism 5 software (San Diego, California, USA) and are expressed as mean ± SEM. Statistical analysis was performed using the paired Student’s *t*-test. *P*-values < .05 were defined as significant.

## Results

### Matrine Could Decrease the Expression Level of Occludin Induced by MicroRNA-155 in Caco-2 Cells

**Figure 1.**
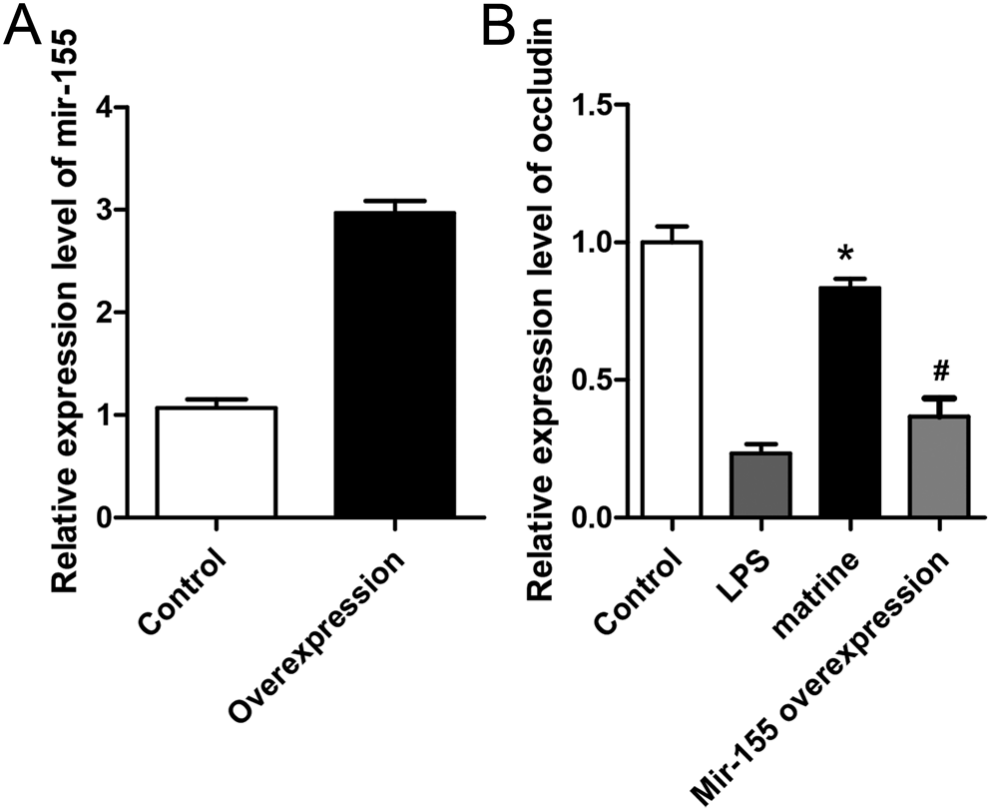
Matrine could attenuate the decrease of the expression induced by microRNA (miR)-155. (A) Analysis of the relative expression level of miR-155 determined by quantitative real-time polymerase chain reaction in miR-155-overexpressed Caco-2 cells and control. (B) LPS could lower the expression of occludin. However, after the pretreatment of matrine, the expression of occludin was increased, while miR-155 overexpression inhibits the effects of matrine. * vs. LPS group, *P* < .05, * vs. #, *P* < .05.

To determine the effect of matrine on the intestinal barrier-associated protein, occludin, the Caco-2 cells were transfected with the miR-155 precursor. Quantitative real-time polymerase chain reaction indicated that the miR-155 expression was significantly increased in the cells transfected with the miR-155 mimic compared with control (*P* < .05, Figure 1A). Lipopolysaccharide (LPS) might inhibit the expression of occludin in Caco-2 cells. However, after the pretreatment of matrine, the occludin expression level was increased, while miR-155 overexpression inhibits the effect of matrine (*P* < .05, Figure 1B).

### Matrine Could Protect the Expression of the Signal of ROCK1, Which Could be Inhibited by MicroRNA-155

**Figure 2.**
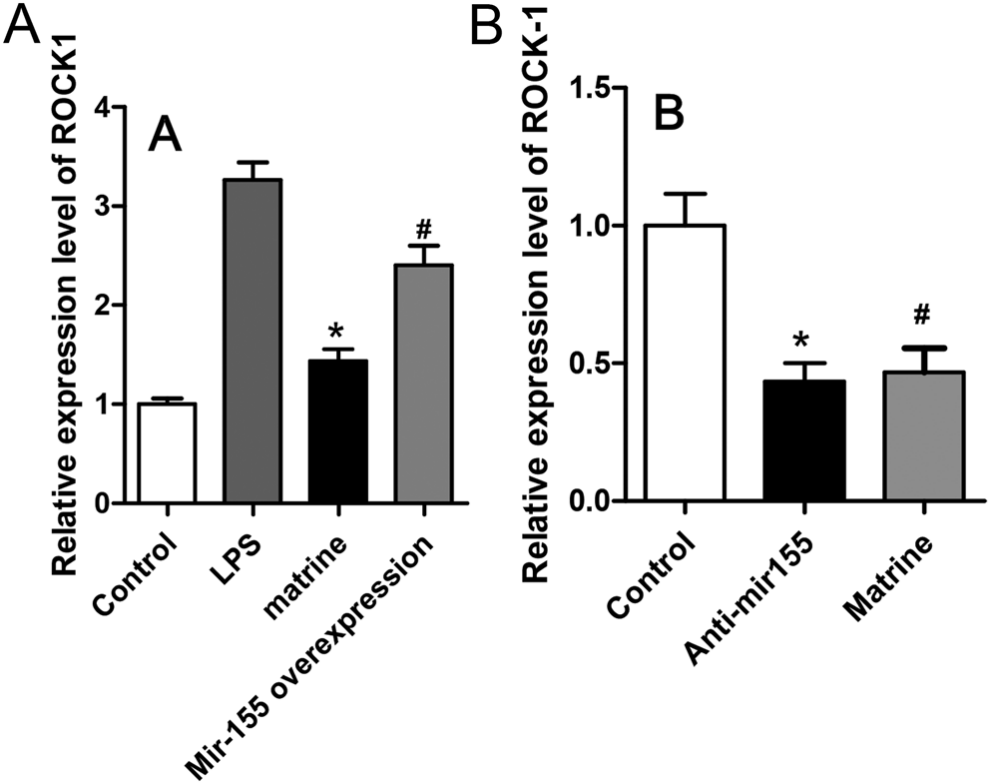
MicroRNA (miR-155) can upregulate ROCK1 signal and inhibit the effects of matrine. (A) LPS could increase the expression of ROCK1 in Caco-2 cells, significantly, and matrine inhibits the effects of LPS; however, after miR-155 was transfected, the ROCK1 expression was upregulated. (B) Analysis of the relative expression level of ROCK1 at the mRNA level in anti-miR-155 transfected Caco-2 cells and control with or without the pretreatment of matrine. * vs. LPS group, *P* < .05, * vs. #, *P* < .05.

To further investigate the molecular mechanism of matrine and miR-155 during the induction of intestinal TJ, we determined the expression level of Rock1 by qRT-PCR after the transfection of miR-155 with or without the pretreatment of matrine. LPS could promote the expression of ROCK1 in Caco-2 cells, significantly, and matrine could inhibit the effect of LPS; however, in the group in which the precursor of miR-155 was transfected, ROCK1 expression was upregulated again (*P* < .05, Figure 2A). Meanwhile, after Caco-2 cells were transfected with miR-155, ROCK1 expression was downregulated with or without the pretreatment of matrine (*P* < .05, Figure 2B).

### Treatment of Dextran Sulfate Sodium-Induced Mice Colitis Confirmed the Relationship Between MicroRNA-155 and ROCK1

**Figure 3.**
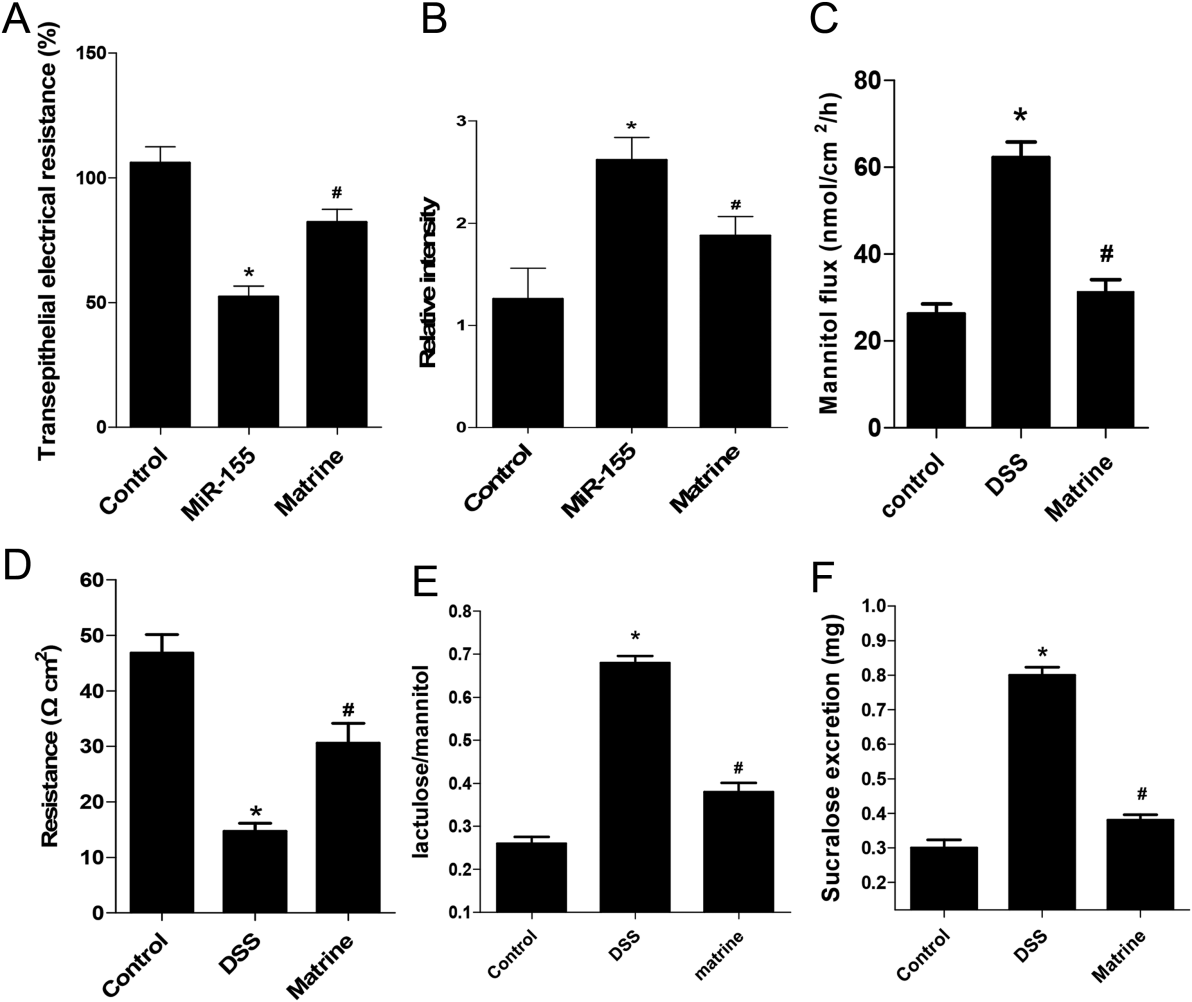
Matrine could lower the intestinal permeability through the inhibition of microRNA (miR)-155 and ROCK1 pathway. (A) Transepithelial electrical resistance was lowered after the miR-155 overexpression in Caco-2 cells, which could be relieved by matrine. (B) The relative intensity was enhanced after the miR-155 overexpression, which could be relieved by matrine. (C) Mannitol flux was increased in the dextran sulfate sodium (DSS)-induced mice colitis group compared with the control wild-type (WT) group, which could be relieved by matrine. (D) The resistance was decreased in the DSS-induced mice colitis group compared with the control WT group, which could be relieved by matrine. (E) The lactulose/mannitol rate was higher in the DSS-induced mice colitis compared with the control WT group at 8- and 12-week mice, which could be relieved by matrine. (F) The sucralose excretion was higher in the DSS-induced mice colitis compared with the control WT group at 8- and 12-week mice *in vivo*, which could be relieved by matrine. * *P* < .05, * vs. #, *P* < .05. Three independent experiments were performed for the cell test, and 15 experiments were performed for mice.

The TER was significantly lower after transfection of miR-155 into Caco-2 cells (*P* < .05, Figure 3A), whereas the relative intensity was enhanced by miR-155; however, pretreatment of matrine could relieve the effect of miR-155 (*P* < .05, Figure 3B). Ussing chamber assay showed a higher permeability of DSS-induced mice colitis compared with the control wild-type (WT) group, while the effects could be relieved by giving the drugs of matrine to mice (*P* < .05, Figure 3C and 3D). The lactulose/mannitol rate and sucralose excretion were used to measure the permeability of small intestine and colon. The lactulose/mannitol rate was higher in the DSS-induced mice colitis compared with the WT mice, which could be relieved by matrine (*P* < .05, Figure 3E). The sucralose excretion was higher in the DSS-induced mice colitis, compared with WT mice, which could be relieved by matrine (*P* < .05, Figure 3E).

**Figure 4.**
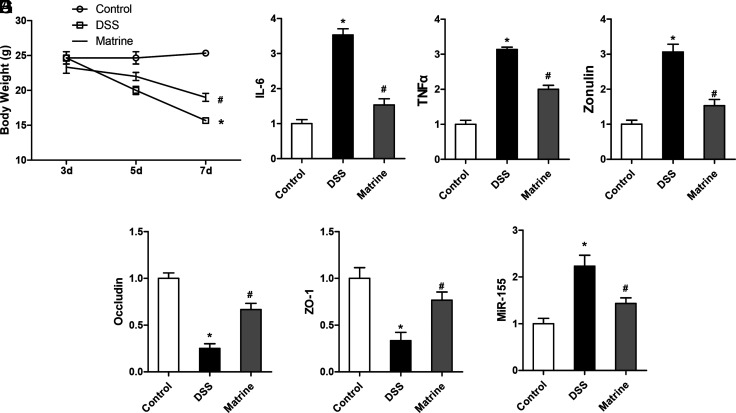
Matrine could relieve the destruction of tight junction. (A) The body weight of dextran sulfate sodium (DSS)-induced mice colitis was lower, while after the pretreatment of anti-ROCK1, the body weight became higher compared with that of the DSS group. (B–D) Interleukin-6 (IL-6) and tumor necrosis factor α (TNF-α) levels were higher in the DSS-induced mice colitis group compared with the wild-type group in mice blood, which could be relieved by anti-ROCK1. (E and F) Occludin and ZO-1 in colon tissues also decreased in the DSS-induced mice colitis group, while the effects could be relieved by giving the drugs of anti-ROCK1 to mice. (G) Matrine could lower the expression of microRNA (miR)-155 in the DSS-induced mice colitis model. *P < .05, * vs. #, *P* < .05. Fifteen independent experiments were performed for mice.

The body weight of DSS-induced mice colitis was significantly lower, compared with control, and matrine could relieve the decrease of body weight significantly (*P* < .05, Figure 4A). The qRT-PCR assays revealed that all serum IL-6, tumor necrosis factor α (TNF-α), and zonulin level at the mRNA levels significantly increased in the DSS-induced mice colitis relative to the WT group, which could be relieved by adding matrine in their drinking water (*P* < .05, Figure 4B–D). We further performed the assay of colon tissues of mice. Determination of occludin and Zonula occludens-1 (ZO-1) also indicated a significant decrease in the DSS-induced mice colitis group, while the effects could be relieved by giving the drugs of matrine to mice (*P* < .05, Figure 4E and 4F). The expression levels of miR-155 were detected as well, and the results indicated that matrine could lower the expression of miR-155 in the DSS-induced mice colitis model (*P* < .05, Figure 4G).

### Low Levels of Occludin and ZO-1 and High Levels of MicroRNA-155, Zonulin, Interleukin-6, and Tumor Necrosis Factor α (Serum) Levels Were Detected in Clinical Stercoral Samples

**Figure 5.**
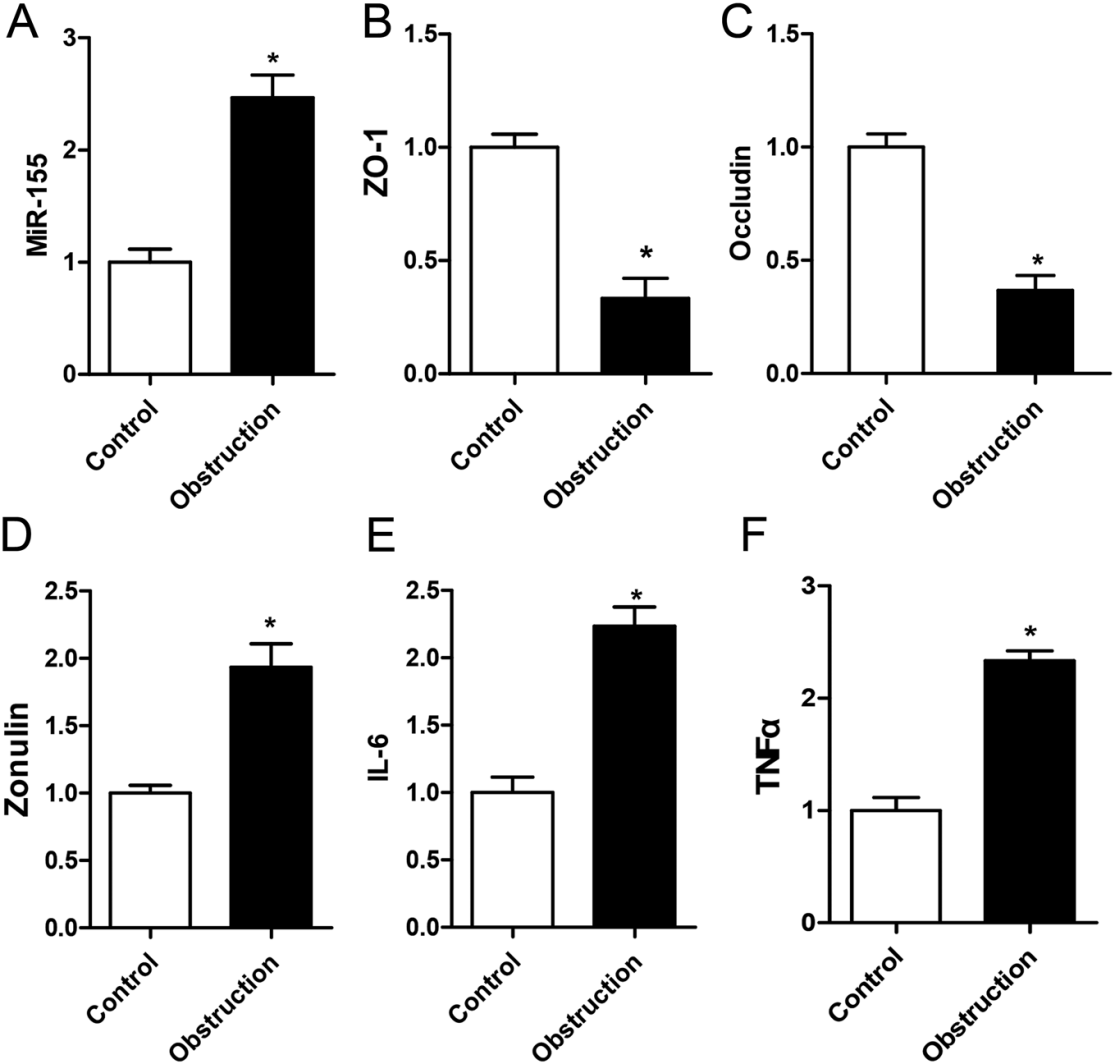
Assay of the clinical samples of acute stercoral obstruction patients to detect the levels of microRNA (miR)-155, occludin, and ZO-1 in the colon tissues and zonulin, interleukin-6 (IL-6), and tumor necrosis factor α (TNF-α) in the serum. (A) The expression levels of miR-155 increased in the stercoral obstruction group compared with the paired obstruction-adjacent normal colon tissues. (B and C) The occludin and ZO-1 expression levels decreased significantly in the stercoral obstruction group compared with that of the paired obstruction-adjacent normal colon tissues. (D–F) Serum levels of zonulin, IL-6, and TNF-α rose in the acute stercoral obstruction group compared with those of the volunteers. *P < .05. Ten independent experiments were performed for each test.

Serum and tissue samples of acute stercoral obstruction patients were used to evaluate the expression level of miR-155. Fresh colon tissues and serum were collected from the stercoral obstruction and paired with adjacent normal colon tissues to determine the expression levels of mR-155 and related inflammatory molecules. Tissue was collected from the Fifth Affiliated Hospital of Guangzhou Medical University, which was approved by the Scientific and Ethical Committee of the Fifth Affiliated Hospital of Guangzhou Medical University (approval no: 201600128) in accordance with approved human subject guidelines. The levels of miR-155 in clinical colon tissues of stercoral obstruction patients were significantly higher than that of adjacent normal colon tissues (*P* < .05, Figure 5A). Meanwhile, the occludin and ZO-1 expression levels declined significantly in clinical colon tissues of stercoral obstruction patients (*P* < .05, Figure 5B and 5C). Serum zonulin, IL-6, and TNF-α levels were determined by the enzyme linked immunosorbent assay (ELISA) kit (RayBiotech, CA, USA) in patients with intestinal barrier dysfunction, which was caused by acute stercoral obstruction. Results showed that higher serum levels of zonulin, IL-6, and TNF-α were found in the acute stercoral obstruction patients than volunteers (*P* < .05, Figure 5D–F).

## Discussion

In this study, we investigated the protective effects of matrine on intestinal barrier via the molecular mechanism of miR-155 in Caco-2 cell lines, DSS-induced mice colitis, and clinical samples of stercoral obstruction patients. Results indicated that matrine could promote the Rho-Rock pathway-related protein ROCK1 in Caco-2 cells and maintain the TJ, while the pretreatment of matrine could inhibit the protective effects of matrine. Determination of mice and clinical samples also showed the protective effects of matrine on TJ and the molecular mechanism and signaling pathway of miR-155 and ROCK1.

In our study, we first transfected the Caco-2 cell line with the miR-155-overexpressed plasmid and found that the expression level of occludin was significantly declined compared with the group of matrine pretreatment. Therefore, we deduced that matrine could protect the intestinal barrier from dysfunction through the miR-155 signal pathway. It is reported that miR-155 contributes to intestinal barrier dysfunction by inhibiting the HIF-1α/Trefoil factor peptide-3 (TFF-3) axis signal.^17^ However, miR-155 has not been reported as the key molecular mechanism during the protection of the intestinal barrier by matrine. Then, the Caco-2 cells were transfected by miR-155 to investigate the regulative effects of matrine and ROCK1. In the group that the precursor of miR-155 was transfected into Caco-2 cells, ROCK1 expression was upregulated compared with the matrine pretreatment group. However, after miR-155 inhibitor was transfected into Caco-2 cells, the expression level of ROCK1 was downregulated. In our previous study, we also found that mir-21 could inhibit the Rho-ROCK pathway by decreasing the ROCK1 expression during the process of intestinal barrier protection.^18^ It is also reported that Rho-ROCK might be an important molecular signal during the regulation of intestinal inflammation, permeability, TJ, and intestinal barrier.^22^ Moreover, we verified the role of matrine and miR-155 in the DSS-induced mice colitis model and clinical samples of stercoral obstruction patients. We further investigated the functions of matrine and miR-155 in the mice with DSS-induced colitis model and clinical serum and tissue samples of stercoral obstruction patients. Our study indicated that miR-155 could be an important signal during the protection of DSS-induced mice colitis by matrine. Clinical samples might indicate that miR-155 and other inflammatory factors were increased in the stercoral obstruction patients group, compared with control, while intestinal barrier-related proteins occludin and ZO-1 were decreased.

It is reported that miR-155 could cause intestinal barrier dysfunction by inducing inflammation and altering gut microecology in the pancreatitis model.^23^ MicroRNA-155 overexpression could increase the intestinal inflammatory factor significantly and decrease the TJ proteins. Another study investigated the microbiota 16S rRNA sequencing and found that miR-155 promoted gut microbiota dysbiosis. Furthermore, they showed that miR155 may regulate intestinal microbiota via the toll-like receptors-4/myeloid differentiation factor 88 (TLR4/MYD88) pathway, leading to serious acute pancreatitis (SAP)-related intestinal injury by the regulation of inflammatory mediators.^23^ Another study showed the association between miR-155 and intestinal barrier dysfunction by nuclear factor (NF)-kappaB.^15^ Mice were injected with an miR-155 inhibitor, which recovered from the intestinal injury. Overexpression of miR-155 increased the NF-kappaB and p-NF-kappaB expression and localization, which could be relieved by adding the miR-155 inhibitor.^15^ Pretreatment with an NF-kappaB inhibitor could improve cell permeability and increase occludin and ZO-1 expression levels. Li et al^24^ showed that miR-155 promoted colitis-associated intestinal fibrosis through the pathway of Hd1 Binding Protein (HBP1)/Wnt/beta-catenin signaling. They reported that the overexpression of miR-155 promoted trinitro-benzene-sulfonic (TNBS)-induced intestinal fibrosis, and HBP1 might be a target of miR-155.^24^

One study showed that matrine significantly lowers the histological score of ulcerative colitis (UC) mice, by decreasing the inflammatory cytokines and maintaining the intestinal barrier function and integrity.^6^ Further study showed that matrine significantly inhibited the PPAR-α-signaling pathway.^6^ Furthermore, matrine could regulate the intestinal microbiota, by regulating *Barnesiella intestinihominis* and *Helicobacter ganmani*.^6^ Another study demonstrated that matrine could decrease intestinal inflammation and enhance the chemokine receptor 7 expression.^25^ Moreover, matrine could alleviate LPS-induced inflammation via downregulating IL-1beta, IL-17, and malondialdehyde expression.^25^ In our study, we mainly investigated the protective effects of matrine via the miR-155-signaling pathway from intestinal barrier dysfunction.

One of the limitations of our study may be that we could not use the clinical intervention of matrine to further verify the protective effects of matrine on intestinal barrier and its impact on miR-155 expression. Whether there are middle molecular mechanisms and pathways should also be studied as well. Further studies or clinical trials might be needed to verify this hypothesis.

## Conclusion

In conclusion, matrine could maintain TJ and protect intestinal barrier from dysfunction. The molecular mechanism may be that matrine might inhibit miR-155 and increase the expression level of TJ proteins.

## Figures and Tables

**Table 1.** PCR Primer

Gene	Primer
ROCK1	(F) 5ʹ-GGTGGTCGGTTGGGGTATTTT-3ʹ
	(R) 5ʹ-CGCCCTAACCTCACTTCCC-3ʹ
IL-6	(F) 5ʹ-CCAGAAGACCAGAGGAAA-3ʹ
	(R) 5ʹ-GGAAATCGTGGAAATGAG-3ʹ
TNF-α	(F) 5ʹ-GACGTGGAACTGGCAGAAGAG-3ʹ
	(R) 5ʹ-TTGGTGGTTTGTGAGTGTGAG -3ʹ
Occludin	(F) 5ʹ-TTGAAAGTCCACCTCCTTACAGA-3ʹ
	(R) 5ʹ-CCGGATAAAAAGAGTACGCTGG-3ʹ
ZO-1	(F) 5ʹ-ACCAGTAAGTCGTCCTGATCC-3ʹ
	(R) 5ʹ-TCGGCCAAATCTTCTCACTCC-3ʹ

IL-6, interleukin-6; PCR, polymerase chain reaction; TNF-α, tumor necrosis factor α.

## References

[1] Bartel DP . MicroRNAs: genomics, biogenesis, mechanism, and function. Cell. 2004;116(2):281 297. (10.1016/s0092-8674(04)00045-5) 14744438

[2] Carthew RW Sontheimer EJ . Origins and mechanisms of miRNAs and siRNAs. Cell. 2009;136(4):642 655. (10.1016/j.cell.2009.01.035) 19239886 PMC2675692

[3] Landgraf P Rusu M Sheridan R , et al. A mammalian microRNA expression atlas based on small RNA library sequencing. Cell. 2007;129(7):1401 1414. (10.1016/j.cell.2007.04.040) 17604727 PMC2681231

[4] Farh KK Grimson A Jan C , et al. The widespread impact of mammalian microRNAs on mRNA repression and evolution. Science. 2005;310(5755):1817 1821. (10.1126/science.1121158) 16308420

[5] Cha ST Tan CT Chang CC , et al. G9a/RelB regulates self-renewal and function of colon-cancer-initiating cells by silencing Let-7b and activating the K-RAS/beta-catenin pathway. Nat Cell Biol. 2016;18(9):993 1005. (10.1038/ncb3395) 27525719

[6] Yao H Shi Y Yuan J Sa R Chen W Wan X . Matrine protects against DSS-induced murine colitis by improving gut barrier integrity, inhibiting the PPAR-alpha signaling pathway, and modulating gut microbiota. Int Immunopharmacol. 2021;100:108091. (10.1016/j.intimp.2021.108091) 34474274

[7] You L Yang C Du Y , et al. A systematic review of the pharmacology, toxicology and pharmacokinetics of matrine. Front Pharmacol. 2020;11:01067. (10.3389/fphar.2020.01067) PMC7526649 33041782

[8] Wu C Xu Z Gai R Huang K . Matrine ameliorates spontaneously developed colitis in interleukin-10-deficient mice. Int Immunopharmacol. 2016;36:256 262. (10.1016/j.intimp.2016.04.038) 27179305

[9] Cheng H Xia B Zhang L , et al. Matrine improves 2,4,6-trinitrobenzene sulfonic acid-induced colitis in mice. Pharmacol Res. 2006;53(3):202 208. (10.1016/j.phrs.2005.11.001) 16332442

[10] Li P Lei J Hu G Chen X Liu Z Yang J . Matrine mediates inflammatory response via gut microbiota in TNBS-induced murine colitis. Front Physiol. 2019;10:28. (10.3389/fphys.2019.00028) PMC6376167 30800071

[11] Liu ZH Huang MJ Zhang XW , et al. The effects of perioperative probiotic treatment on serum zonulin concentration and subsequent postoperative infectious complications after colorectal cancer surgery: a double-center and double-blind randomized clinical trial. Am J Clin Nutr. 2013;97(1):117 126. (10.3945/ajcn.112.040949) 23235200

[12] Liu T Liang X Lei C , et al. High-fat diet affects heavy metal accumulation and toxicity to mice liver and kidney probably via gut microbiota. Front Microbiol. 2020;11:1604. (10.3389/fmicb.2020.01604) PMC7399142 32849333

[13] Jiang Z Yang F Qie J , et al. TNF-alpha-induced miR-21-3p promotes intestinal barrier dysfunction by inhibiting MTDH expression. Front Pharmacol. 2021;12:722283. (10.3389/fphar.2021.722283) PMC8415152 34483933

[14] Wang Z Zhong C Cao Y , et al. LncRNA DANCR improves the dysfunction of the intestinal barrier and alleviates epithelial injury by targeting the miR-1306-5p/PLK1 axis in sepsis. Cell Biol Int. 2021;45(9):1935 1944. (10.1002/cbin.11633) 34003569

[15] Cao YY Wang Z Wang ZH Jiang XG Lu WH . Inhibition of miR-155 alleviates sepsis-induced inflammation and intestinal barrier dysfunction by inactivating NF-kappaB signaling. Int Immunopharmacol. 2021;90:107218. (10.1016/j.intimp.2020.107218) 33296782

[16] McKenna LB Schug J Vourekas A , et al. MicroRNAs control intestinal epithelial differentiation, architecture, and barrier function. Gastroenterology. 2010;139(5):1654 1664. (10.1053/j.gastro.2010.07.040) 20659473 PMC3156097

[17] Liu Y Zhu F Li H , et al. MiR-155 contributes to intestinal barrier dysfunction in DSS-induced mice colitis via targeting HIF-1alpha/TFF-3 axis. Aging (Albany NY). 2020;12(14):14966 14977. (10.18632/aging.103555) 32713852 PMC7425479

[18] Liu Z Li C Chen S , et al. MicroRNA-21 increases the expression level of occludin through regulating ROCK1 in prevention of intestinal barrier dysfunction. J Cell Biochem. 2019;120(3):4545 4554. (10.1002/jcb.27742) 30302792

[19] Liu Z Kang L Li C , et al. Knockout of MIMP protein in lactobacillus plantarum lost its regulation of intestinal permeability on NCM460 epithelial cells through the zonulin pathway. BMC Gastroenterol. 2014;14:171. (10.1186/1471-230X-14-171) PMC4287571 25277875

[20] Song L Liu Z Hu HH , et al. Proto-oncogene Src links lipogenesis via lipin-1 to breast cancer malignancy. Nat Commun. 2020;11(1):5842. (10.1038/s41467-020-19694-w) PMC7672079 33203880

[21] Onyeagucha BC Mercado-Pimentel ME Hutchison J Flemington EK Nelson MA . S100P/RAGE signaling regulates microRNA-155 expression via AP-1 activation in colon cancer. Exp Cell Res. 2013;319(13):2081 2090. (10.1016/j.yexcr.2013.05.009) 23693020 PMC3726211

[22] Marchiando AM Shen L Graham WV , et al. The epithelial barrier is maintained by in vivo tight junction expansion during pathologic intestinal epithelial shedding. Gastroenterology. 2011;140(4):1208-1218. (10.1053/j.gastro.2011.01.004) PMC3066304 21237166

[23] Yang X Wan J Li N , et al. MiR155 disrupts the intestinal barrier by inducing intestinal inflammation and altering the intestinal microecology in severe acute pancreatitis. Dig Dis Sci. 2022;67(6):2209 2219. (10.1007/s10620-021-07022-1) 34341909

[24] Li N Ouyang Y Xu X Yuan Z Liu C Zhu Z . MiR-155 promotes colitis-associated intestinal fibrosis by targeting HBP1/Wnt/beta-catenin signalling pathway. J Cell Mol Med. 2021;25(10):4765 4775. (10.1111/jcmm.16445) 33769664 PMC8107084

[25] Wu G Zhou W Zhao J , et al. Matrine alleviates lipopolysaccharide-induced intestinal inflammation and oxidative stress via CCR7 signal. Oncotarget. 2017;8(7):11621 11628. (10.18632/oncotarget.14598) 28086227 PMC5355291

